# The effect of mother’s age on the neonatal cord serum’s oxidative stress index and maternal and neonatal outcomes: a case control study

**DOI:** 10.1186/s12884-023-06239-4

**Published:** 2024-01-12

**Authors:** Fatemeh Imanparast, Bahman Hashemi, Fatemeh Mokhtari, Pegah Mohaghegh, Fereshteh Farzan Azar, Fatemeh Mehvari

**Affiliations:** 1 Department of Biochemistry and Genetics, Faculty of Medicine, Arak University of Medical Sciences, Arak, Iran; 2 Department of Midwifery, Arak Branch, Islamic Azad University, Arak, Iran; 3 Community and preventive medicine specialist, Department of community medicine, Faculty of medicine, Arak University of Medical Sciences, Arak, Iran; 4 Department of Midwifery, Faculty of Medicine, Arak University of Medical Sciences, Arak, Iran

**Keywords:** Total antioxidant capacity, Total oxidant status, Infant outcomes, Maternal outcomes, Aging

## Abstract

**Background:**

One of the main challenges of many societies in reducing and ageing of the population is marriage at an advanced age in women and decrease of producing offspring due to the concern of increasing the probability of maternal and neonatal outcomes. The mother’s oxidative stress conditions during pregnancy affect mothers and their baby’s health. Aging is one of the increasing factors of oxidants in the body. Aim of this study is the compartion total antioxidant capacity (TAC), total oxidants status (TOS), oxidative stress index (OSI) values, and maternal and neonatal outcomes in three groups of mothers with different age ranges from 20 to 29, 30 to 34, and 35 to 45 years old.

**Methods:**

164 pregnant women were grouped according to age into three groups: 25 to 30 (group I), 30 to 35 (group II), and 35 to 45 years old (group III). The umbilical cord blood samples were taken to the assay TAC, TOS, and OSI (TOS/TAC**).** The Kolmogorov-Smirnov test was employed to assess the normal distribution of countinus variables. The one-way ANOVA and Kruskal-Wallis test were used to compare anthropometric and biochemical factors between groups.

**Results:**

TAC levels decreased non-significantly (438.2 ± 102; 431.7 ± 99.8; and 428.2 ± 100.26 for groups I, II, and III respectively, *P* value = 0.99), TOS levels increased significantly (23.93 ± 11.7; 25.4 ± 12.3; and 28.2 ± 12.7 for groups I, II, and III respectively, *P* value = 0.034), and OSI increased non-significantly with increasing maternal age (0.055 ± 0.044; 0.091 ± 0.031; 0.069 ± 0.005, for groups I, II, and III respectively, *P* value = 0.14). Increasing age did not significantly affects the maternal and infant birth outcomes.

**Conclution:**

The results showed that the increasing the age of the mother up to 45 doesn’t have a significant effects on the value of OSI and the maternal and infant outcomes.

## Background

Research has shown the near and inextricable interconnection between maternal and newborn health. Various factors can threaten this health, lead to maternal and newborn morbidity, and induce mortality and adverse outcomes in both [1, 2].

It is noteworthy that reactive oxygen species (ROS) have the positive role in the process of fertilization and fetal development, but this is in a situation where the body’s antioxidant defense is sufficient [3, 4]. Optimal ROS is essential in regulating placental angiogenesis for a successful placental vasculature. Also, it is necessary for an adequate exchange of nutrients and oxygen between mother and fetus [5]. Antioxidants play a significant role in protecting against molecular oxidative damage. They contain the general endogenous antioxidant and exogenous system that counteract free radicals and neutralize oxidants [4].

One of the important mechanisms affecting the health of the fetus and the newborn is the mother’s oxidative stress (OS) conditions during pregnancy and childbirth. High metabolic activity in the fetoplacental compartment introduces pregnancy as a state of oxidative stress, which is considered a risk factor during pregnancy. Studies show that oxidative stress in fetal body structure activates the sequence of genes involved in inflammation, coagulation, fibrinolysis, and the cell cycle. Under special conditions, the production of free radicals overcomes the body’s antioxidant defenses. Also, these radicals destroy tissues and organs of the body (e.g., the lungs and brain), thereby threatening the babies’ life and affecting their quality of life. Studies have shown that oxidative stress is involved in developing neonatal diseases such as bronchopulmonary dysplasia, preterm neonatal retinopathy, and neonatal enterocolitis [6–8].

Aging is associated with a progressive reduction in the efficiency of biochemical and physiological processes and increased susceptibility to disease. One of the main reasons for these unfavorable changes and age-related disease is oxidative stress (OS) conditions [9, 10].

One of the major challenges in our countray is the birth rate decline due to the increase in the marriage age and the decrease in the tendency to have children at an older age for various reasons, including fear of increasing the consequences for mother and child in older mothers [11].

In this study, we hypothesized that increasing maternal age increases oxidative stress index (OSI) of newborn cord serum and maternal and newborn birth outcomes. Therefore, we compared total antioxidant capacity (TAC), total oxidant status (TOS), OSI values and maternal and neonatal outcomes in three groups of mothers with different age ranges from 20 to 29, 30 to 34, and 35 to 45 years.

## Materials and methods

### Study design and participants

This case control study was conducted at the Maternity ward of Taleqani Hospital in Arak, Iran, from January 2022 to September 2022. 164 healthy pregnant mothers participiated in this study in the age range of 20 to 45 years in 3 age groups of 20 to 29, 30 to 34, and 35 to 45 years, respectively.

Maternal age was considered a risk factor, and TAC, TOS, and OSI (oxidative stress index) in neonatal cord blood and outcomes were assessed.

### The inclusion criteria

The inclusion criteria were pregnant mothers with normal delivery conditions, the first delivery age of 25–45 years, BMI < 30 kg/m^2^, normal activity, diet, and mental and psychological conditions at delivery.

### The exclusion criteria

The exclusion criteria were mothers with diabetes (pregnancy), preeclampsia, chronic inflammatory diseases, heart disease, liver-kidney disease, thyroid disease, and other endocrine diseases. Also, mothers who smoke, have severe vitamin deficiency during pregnancy, and with mental health problems during pregnancy were discarded.

### Sample size and sampling

To estimate the sample size, to compare the mean TAC in the two age groups of mothers < 35 years and ≥ 35 according to the Enoch Odame Anto study, the mean TAC in mothers over 35 years of age were 0.7 mmol / L and mothers under 35 years of age were 1.5 mmol / L [12] according to the formula for comparing the means in two independent groups,the sample size of our study consisted of 164 healthy pregnant mothers in the age range of 20 to 45 years in 3 age groups of 20 to 29, 30 to 34, and 35 to 45 years, respectively.Also, we considered type-1 (α) and type-2 errors (β) of 0.05 and 0.20 (power = 80%), respectively. Convenience sampling was done.

### Instruments and data collection procedures

#### Data analysis

The mean ± Standard Deviation, as well as a number (%), were employed to describe the data. The Kolmogorov-Smirnov test was employed to assess the normal distribution of countinus variables. Besed on Central Limit Theorem (sample size is ≥ 30 in each group) The one-way ANOVA test were used to compare Anthropometric and Biochemical factors between groups. All statistical analyses were performed using SPSS version 17 (SPSS, Chicago, IL, USA).

#### Ethical and safety considerations

Ethical approval for this study was obtained from the Committee on Human Research, Publication and Ethics at Arak University of Medical Sciences, Arak, Iran (IR.ARAKMU.REC.1399.6104). We confirm that all experiments were performed in accordance with relevant guidelines and regulations. All mothers completed a written informed consent form and signed/thumb-printed.

#### Biochemical assessments

The umbilical cord blood samples were taken, and the aliquot samples of serums were saved after centrifugation (20 min, 3000 rpm) at -80^˚^C. TAC and TOS were measured by a Kiazist colorimetric kit, Hamedan, Iran. After adding the reagents of the colorimetric kit to 30 µl of the serum sample according to the instructions of the kit, the absorbance of the colored solutions obtained for TAC and TOS was read by a microreader at 530 and 593 μm, respectively.

The OSI was defined as the ratio of the TOS to TAC.

Consequences at birth include Apgar score, infant weight, and presence or absence of respiratory access in the infant were extracted from the mother file and recorded in the checklist.

## Results

This study was conducted from January 2022 to September 2022. The research population consisted of 3 age groups: 20 to 29 (group II), 30 to 34 (group II), and 35–45 years (group III).

Table 1 shows the relationship between TAC, TAS, and OSI. As can be seen, TAC decreased with increasing the mother’s age, but there was no significant relationship between TAC and maternal age in 3 age groups of mothers. In addition, there was a significant direct relationship between TOS and maternal age in 3 age groups of mothers (23.93 ± 11.7, 25.4 ± 12.3, and 28.4 ± 12.7, respectively; *P*-value = 0.034). Finally, there was no significant relationship between OST and maternal age in the 3 age groups of mothers. However, a non-significant increase was identified in group 3 compared to the other two groups.

**Table 1:** comparision of TAC, TOS, and OSI in 3 age groups of mothers

Mean ± SD	Group I (20–29 years) *n* = 59	Group II (30–34 years) *n* = 41	Group III (35-45years) *n* = 65	***P*** value
**TAC (nmol/l)**	438.2 ± 102	431.7 ± 99.8	428.2 ± 100.26	0.99
**TOS (nmol/l)**	23.93 ± 11.7	25.4 ± 12.3	28.2 ± 12.7	0.034
**OSI (TOS/TAC)**	0.055 ± 0.044	0.091 ± 0.031	0.069 ± 0.005	0.140

**ANOVA Test. TAC**: Total antioxidant capacit**y, TOS**: Total oxidants statues, **OST**: Oxidative stress indices

Table 2 shows the relationship between birth and maternal outcomes (quantitative variables including baby weight, baby height, baby head circumference, Score Apgar, Preterm delivery, Abnormal bleeding, Water bag rupture, Stillbirth, Asphyxia, mother weight) and maternal age. There was no significant relationship between these outcomes and maternal age in the 3 age groups.

**Table 2:** The comparision of birth and maternal outcomes (quantitative variables) and maternal age

Mean ± SD	Group I (20–29 years) *n* = 59	Group II (30–34 years) *n* = 41	Group III (≥ 35years) *n* = 65	***P***-value
**Baby weight (gr)**	3720.85 ± 55.08	3175.98 ± 331.5	3219.2 ± 498.2	0.35
**baby height (cm)**	50.8 ± 2.99	50.39 ± 3	51.34 ± 2.84	0.55
**baby head circumference(cm)**	35.2 ± 2.74	34.86 ± 1.3	35.2 ± 2.22	0.14
**Score Apgar**	9 ± 0.18	9.00 ± 00.0	8.94 ± 0.3	0.247
**Preterm delivery (%)**	0.00	1.9	1.2	0.087
**Abnormal bleeding (%)**	5.5	4.3	3	0.12
**Water bag rupture (%)**	4.2	4.8	2.4	0.11
**Stillbirth (%)**	1.2	0.6	1.2	0.96
**Asphyxia (%)**	0.00	0.00	0.00	
**mother weight(kg)**	72.05 ± 12	75.83 ± 11.29	73.9 ± 12.5	0.38

**ANOVA**

## Discussion

Aim of this study is the compartion total antioxidant capacity (TAC), total oxidants status (TOS), oxidative stress index (OSI) values, and maternal and neonatal outcomes in three groups of mothers with different age ranges from 20 to 29, 30 to 34, and 35 to 45 years old. TAC and OSI levels did not change significantly with increasing maternal age, but the amount of TOS increased significantly with increasing maternal age. Also, in this study, increasing age did not significantly affects maternal and infant birth outcomes such as Apgar score, infant weight, stillbirth, and asphyxia. One of the factors in the effectiveness of the maternal age on the TAC level may be the cultural and educational factors of older mothers. Therefore, they need a healthier lifestyle and nutrition and must be monitored during pregnancy. Although TOS increased due to aging, regarding the importance and more care for mothers in old age, these mothers did not show a significant decrease in TAC compared to mothers with younger ages. The results of this study are consistent with some studies and contradictory with some others.

Increased serum levels of oxidants and decreased levels of TAC were associated with intrauterine growth restriction, placenta praevia, abruption placenta, stillbirth, and postpartum hemorrhage (PPH) [13]. Some researchers have reported an increased rate of adverse pregnancy outcomes in women older than 35 years, while others have failed to find any association between advanced maternal age and adverse perinatal outcomes [14, 15]. Myrskylä and Fenelon (2012) found a direct relationship between maternal age and neonatal health outcomes. They explained this association with the effect of aging on the quality of oocytes (eggs) [14]. According to Gila-Díaz et al. (2020), maternal age was not statistically correlated with the TAC of breast milk [16]. Kahveci et al. (2018) showed a significant association between advanced maternal age and gestational diabetes, gestational hypertension, preeclampsia, increased cesarean section rates, and spontaneous late preterm delivery [17]. Anto et al. (2018) reported that increasing maternal age is associated with negative pregnancy outcomes and introduced oxidative stress among the contributing factors [12]. In this study, with increasing maternal age, TAC and oxidative markers decreased and increased, respectively. The increase in oxidative stress is consistent with the results of the present studies; however, TAC did not decrease in older mothers. One of the reasons for this difference probably is more attention to consuming foods containing more antioxidants in the older mothers than the younger mothers. In recent years, in Iran, the mothers who become pregnant for the first time at an older age often have higher levels of education and well-being due to employment, leading to better nutrition during pregnancy. Although the level of oxidants has increased with increasing the maternal age, due to better nutrition and mental conditions of these mothers, the total level of antioxidants in these mothers compensates for and prevents further consequences at birth for them and their infants.

Overall, maybe, the adverse effects of increasing the level of oxidants due to the aging of the mother can be mitigated by improving the nutritional status, using supplements, and providing favorable mental and psychological conditions during pregnancy.

### Limitations of the study

Limitations of this study included a lack of matching of factors such as the accurate BMI, education, diet, and lifestyle of mothers, smoking, medicine use, stress, and restricting participants to only one hospital and one city, and limited outcomes such as colic, allergies, asthma, and digestive problems. It is suggested to perform the same study with a large sample size in several cities. This way, it is possible to incorporate further consequences at birth and in the baby’s first year (including colic, allergies, asthma, and digestive problems). Furthermore, recording mothers’ nutritional conditions and physical movements is recommended.

#### Conclusions

The results showed that the increasing the age of the mother up to 45 doesn’t have a significant effects on the value of OSI and the maternal and infant outcomes.

## Data Availability

All data used in the current study are available from the corresponding author on reasonable request.
